# Internal jugular vein stenosis associated with elongated styloid process: five case reports and literature review

**DOI:** 10.1186/s12883-019-1344-0

**Published:** 2019-06-04

**Authors:** Min Li, Yuan Sun, Chong Ching Chan, Chunqiu Fan, Xunming Ji, Ran Meng

**Affiliations:** 1 Department of Neurology, Xuanwu Hospital, Capital Medical University, 45 Changchun Road, Xicheng District, Beijing, 100053 People’s Republic of China; 2 Department of General Practice, Xuanwu Hospital, Capital Medical University, 45 Changchun Road, Xicheng District, Beijing, People’s Republic of China; 3 Department of Medicine, Queen Elizabeth Hospital, 30 Gascoigne Road, Kowloon, Hong Kong SAR, China; 4 Department of Neurosurgery, Xuanwu Hospital, Capital Medical University, 45 Changchun, Road, Xicheng District, People’s Republic of China

**Keywords:** Internal jugular vein stenosis, Elongated styloid process, Eagle syndrome, Styloid compression

## Abstract

**Background:**

Internal jugular vein stenosis (IJVS), characterized by a series of clinical manifestations, such as head and neck symptoms, visual and ear symptoms, as well as sleep disorder, has been receiving attention in recent years. However, its’ etiologies are not fully understood.

**Case presentation:**

We report a cases series of IJVS induced by styloid oppression. We define it as the stylo-jugular type of Eagle syndrome (ES).

**Conclusions:**

Our study reveals that external oppression, especially by styloid process, is an important etiology of IJVS. The stylo-jugular ES diagnosis can be identified by Computed tomography venography. Whether stylo-jugular ES can be corrected by styloidectomy requires further investigation.

## Background

Internal jugular vein stenosis (IJVS) are characterized as a series of non-specific symptoms, including head symptoms (headache, head noise, dizziness and memory decline), eye symptoms (eye bloating, diplopia, blurred vision and visual field defect), ear symptoms (tinnitus and high-frequency hearing decline), neck discomfort, or sleeping disorder [1–9]. Hence, IJVS does not draw enough attention by both patients and doctors, which often results in misdiagnosis or missed diagnosis. Moreover, the etiologies of IJVS are not fully understood. To the best of our knowledge, venous wall is thinner than that of artery, which lacks smooth muscle and elastic fibers, whereby, veins are more vulnerable to deform under extrinsic compression [10]. Therefore, external structures oppression may be one of the most important etiologies of IJVS. Herein, we describe a cases series of styloid oppression-induced IJVS on the aspects of clinical characteristics, diagnosis and treatment, to give a reference in clinical practice.

## Case presentation

### Case 1

A 65-year-old female complained headache, tinnitus and eye discomfort and even blurred vision consisted for 10 years. Four months ago, she felt a hearing loss in her left ear. Comorbid medical issues included the history of diabetes mellitus, coronary heart disease, sleep apnea syndrome, and lumbar disc herniation and sinusitis surgery. Physical examination showed left ear hearing loss, the pinprick and vibration feelings mildly diminished in bilateral glove-and-stocking territories.

**Fig. 1:**
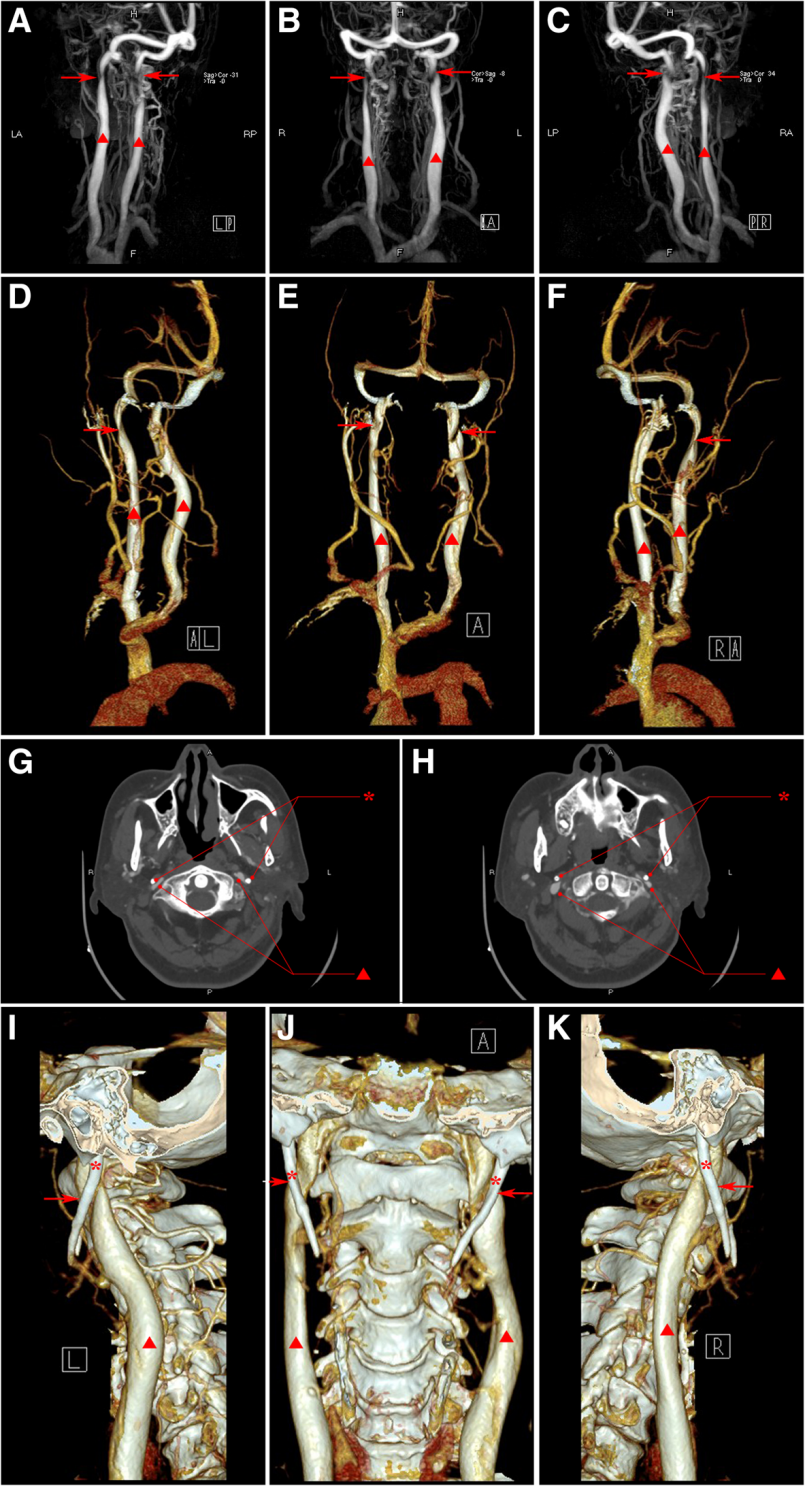
Images of Contrast-MRV (**a-c**), 3D-CTV (**d-f**), axial CTV (**g-h**) and 3D-CTV with bone remodeling (**i-k**) in case 1. The red asterisk represents styloid process, the red triangle represents internal jugular vein and the red arrow indicates the focal stenosis

No positive findings identified in the image of brain computed tomography (CT), magnetic resonance imaging (MRI), as well as trans-cranial color Doppler (TCCD). Carotid ultrasound revealed intima-media thickening and plaques in bilateral carotid arteries. Computed tomography angiography (CTA) revealed arteriosclerosis in head and neck without significant stenosis. Contrast-magnetic resonance venography (MRV) identified bilateral IJVS in J3 segment accompanied with distorted and dilated vertebral venous plexus (Fig. 1a-c). Three-dimensional (3D)-CTV images showed the stenosis at J3 segment of bilateral internal jugular vein (IJV) (Fig. 1d-f). The axial computed tomography venography (CTV) images (Fig. 1g-h) and 3D-CTV images with bone remodeling (Fig. 1i-k) indicated that elongated styloid process compressed bilateral IJV against the transverse process of C1 vertebra. The styloid oppression-induced IJVS in bilateral J3 segment was also identified by Digital subtraction angiography (DSA), and cerebral venous sinuses and IJV thrombi were excluded by Black-blood thrombus image (BBTI).

After she underwent xueshuantong (panax notoginseng saponins) 450 mg (mg)/ intravenous/ daily for 10 days, and aspirin 100 mg/ per oral/ daily and rosuvastatin 10 mg/ per oral/ qn. for 30 days, her symptoms were not improved.

### Case 2

A 58-year-old male complained intermittent dizziness, accompanied with insomnia for 3 years. He developed intermittent headache and numbness of scalp 3 months ago. Comorbid medical issues included the history of hypothyroidism. No positive findings were in his neurological examination.

**Fig. 2:**
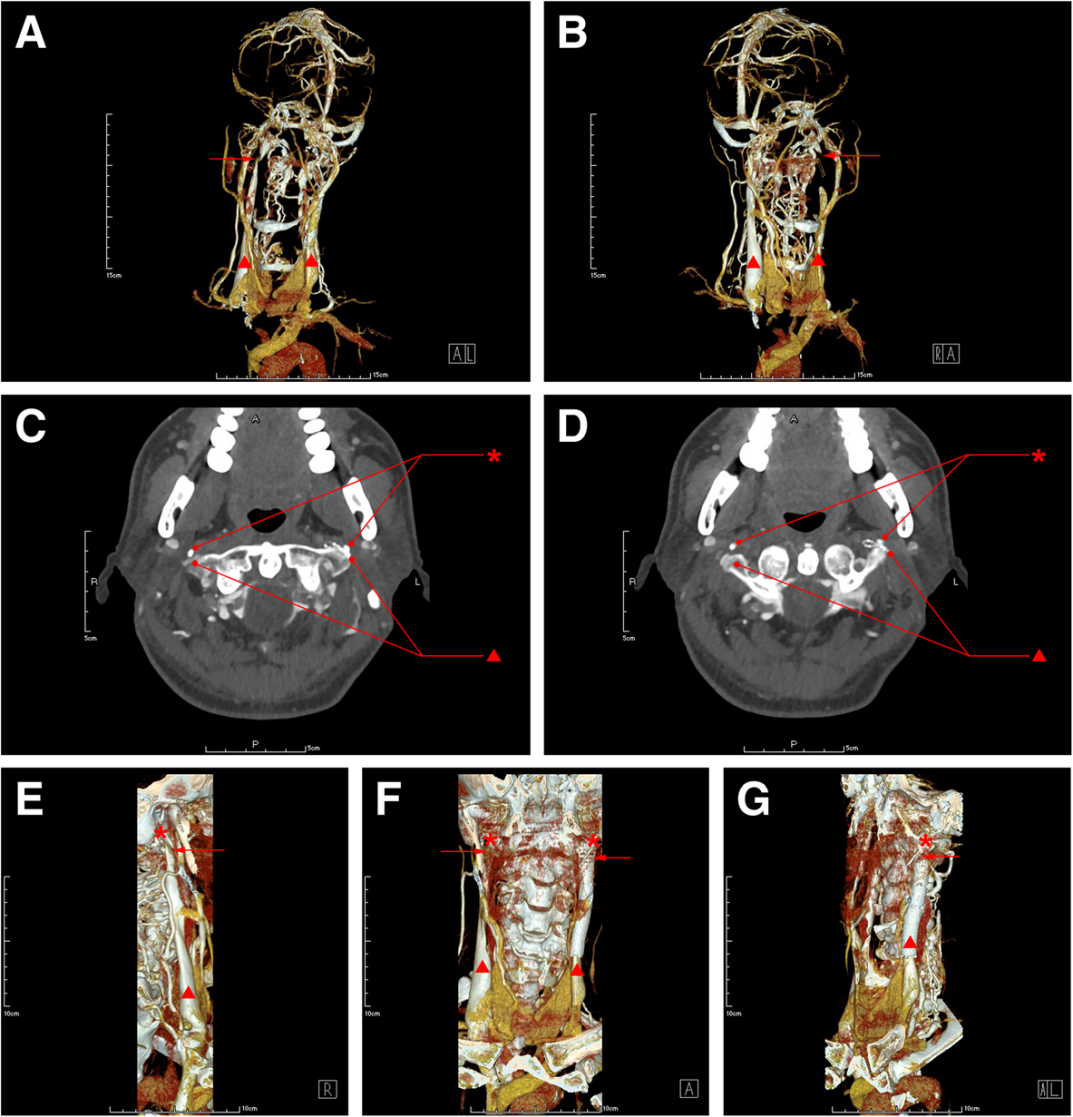
Images of 3D-CTV (**a-b**), axial CTV (**c-d**) and 3D-CTV with bone remodeling (**e-g**) in case 4. The red asterisk represents styloid process, the red triangle represents internal jugular vein and the red arrow indicates the focal stenosis

**Fig. 3:**
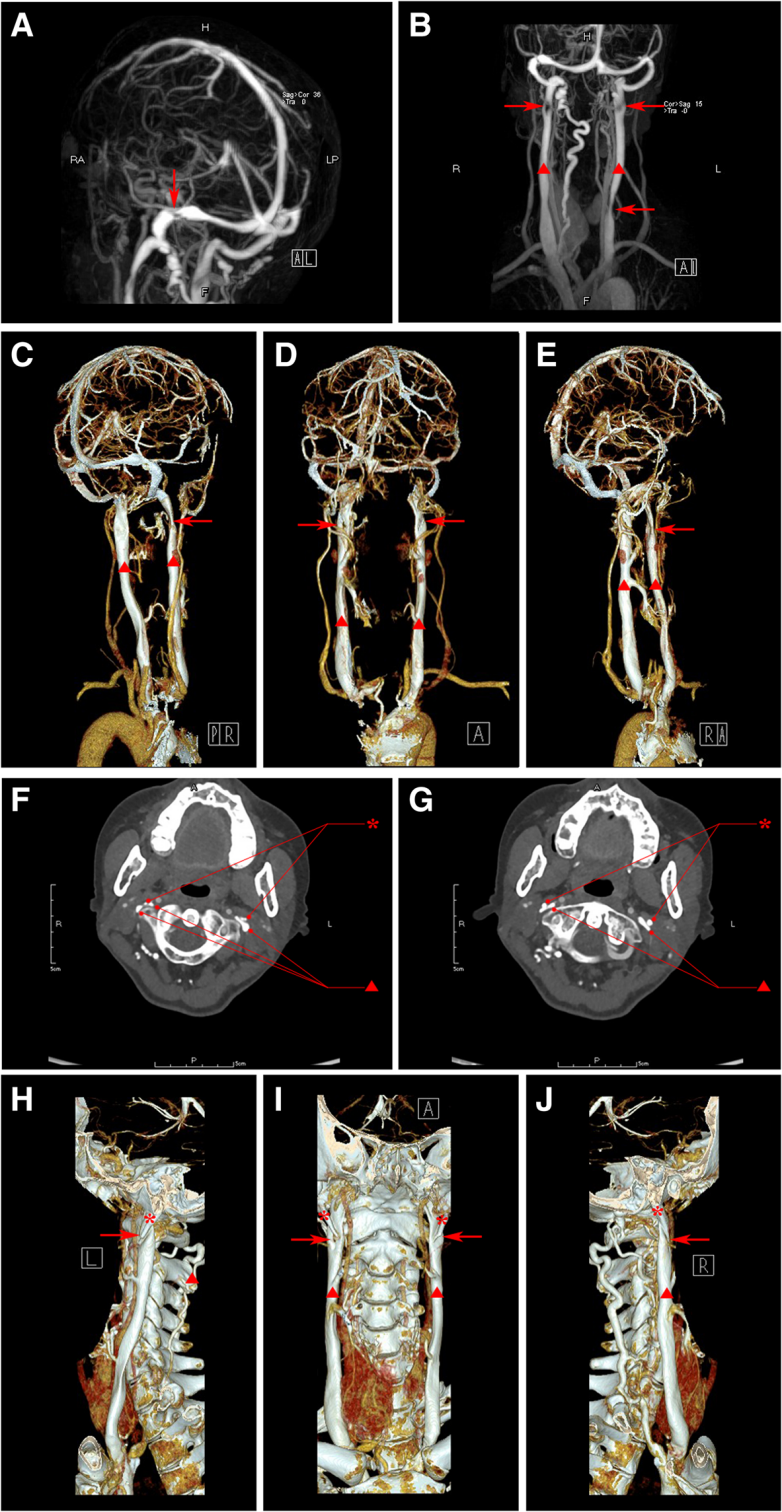
Images of Contrast-MRV (**a-b**), 3D-CTV (**c-e**), axial CTV (**f-g**) and 3D-CTV with bone remodeling (**h-j**) in case 3. The red asterisk represents styloid process, the red triangle represents internal jugular vein and the red arrow indicates the focal stenosis

Brain MRI showed ischemic focus in bilateral subfrontocortical region. Carotid ultrasound and CTA showed mild arteriosclerosis. Contrast-MRV showed focal stenosis at J2-J3 segment of the right IJV and J3 segment of right IJV, accompanied with dilated vertebral venous plexus (Fig. 2a-b). 3D-CTV showed stenosis at bilateral IJV-J3 segment (Fig. 2c-d) and dilated vertebral venous plexus. Axial CTV (Fig. 2e-f) and 3D-CTV with bone remodeling (Fig. 2g-i) showed J3 segment of bilateral IJV was compressed by styloid process and transverse process of C1 vertebra.

He was treated with xueshuantong (panax notoginseng saponins) 450 mg/ intravenous/ daily and alprostadil 10 μg/ intravenous/ daily for 10 days, combined with betahistine 6 mg/ per oral/ daily and pitavastatin 4 mg/ per oral/ qn. for 3 months. All his symptoms were not attenuated.

### Case 3

A 61-year-old female complained insomnia for 10 years. She had no other past medical history. No positive findings were in her neurological examination.

CTA showed mild arteriosclerosis in the head and neck. Jugular ultrasound revealed malformation of right IJV-J3 segment. Contrast-MRV identified the stenosis at the junction of right transverse sinus and sigmoid sinus, and the superior and inferior segment of the left IJV, dysplasia of the superior segment of the right IJV and dilated right vertebral venous plexuses (Fig. 3 a-b). 3D-CTV indicated the stenosis at the junction of right transverse sinus and sigmoid sinus, and at J3 segment of the bilateral IJV (Fig. 3c-e). Axial CTV images (Fig. 3f-g) and 3D-CTV with bone remodeling (Fig. 3h-j) showed the styloid oppression on bilateral IJV-J3 segments against the transverse process of C1 vertebra.

After underwent xueshuantong (panax notoginseng saponins) 450 mg/ intravenous/ daily for 10 days and aspirin 100 mg/ per oral/ daily for 30 days, all her symptoms were not attenuated.

### Case 4

A 60-year-old male patient is with a stridulous tinnitus existed continuously in both earsand hearing decline in left ear for 2 years. He has no other past medical history. Neurological examination is negative except for hearing decline in both ears.

Brain MRI images revealed no positive finding. Lumbar puncture showed the intracranial pressure (ICP) was 200mmH2O. Jugular vein ultrasound identified the elongated venous valve and focal stenosis in left IJV-J2/ J3 segment and right IJV-J3 segment.

The catheter venography indicated the trans-stenotic pressure gradient was 80mmH2O. Two stents (sinus-SuperFlex 10*60) were placed at the stenotic segment in left IJV-J3. DSA showed the corrected blood reflow after stenting. Jugular vein ultrasound after stenting prior to discharge showed normal blood flow in left IJV and focal stenosis in right IJV-J3 segment. The patient then was treatment with warfarin 3 mg/ per oral/ daily combined with aspirin 100 mg/ per oral/ daily for one year and his symptoms were completely disappeared at 19 days followed-up after stenting.

**Fig. 4:**
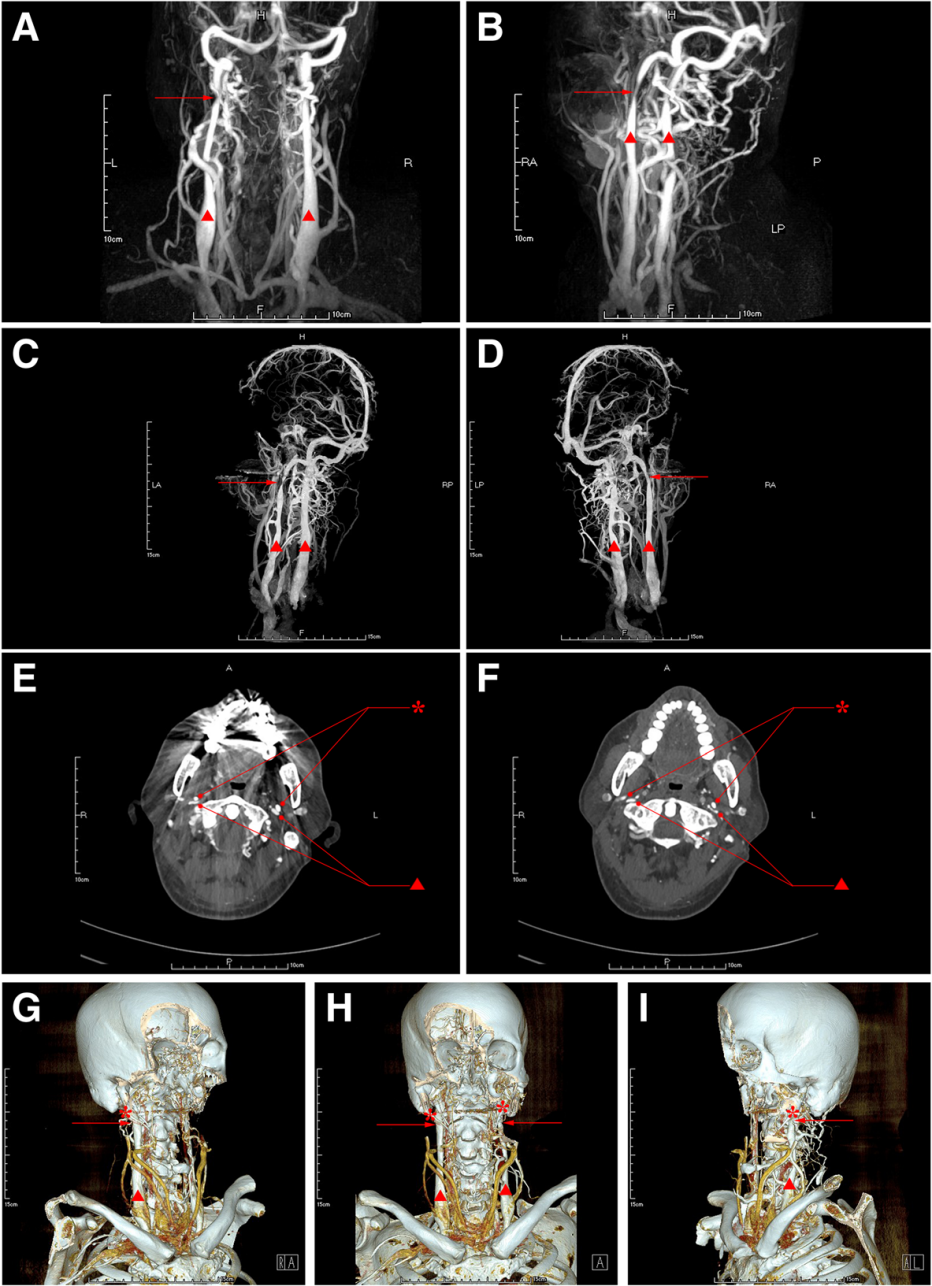
Images of Contrast-MRV (**a-b**), 3D-CTV (**c-d**), axial CTV (**e-f**) and 3D-CTV with bone remodeling (**g-i**) in case 2. The red asterisk represents styloid process, the red triangle represents internal jugular vein and the red arrow indicates the focal stenosis

However, his symptoms reoccurred at the 20th day after stenting. Jugular vein ultrasound showed restenosis in left IJV-J3 segment and focal stenosis in right IJV J3 segment. 3D-CTV revealed the stenosis in bilateral IJV-J3 segment (Fig. 4a-b). Axial CTV images (Fig. 4c-d) and 3D-CTV (Fig. 4e-g) with bone remodeling revealed the bilateral IJV J3 segment was compressed by elongated styloid process and transverse process of C1 vertebra. Although given advices, he refused to receive styloidectomy.

### Case 5

A 49-year-old male complained intermittent dizziness for 3 months. His past medical history included hypertension. No positive findings were in his neurological examination.

**Fig. 5:**
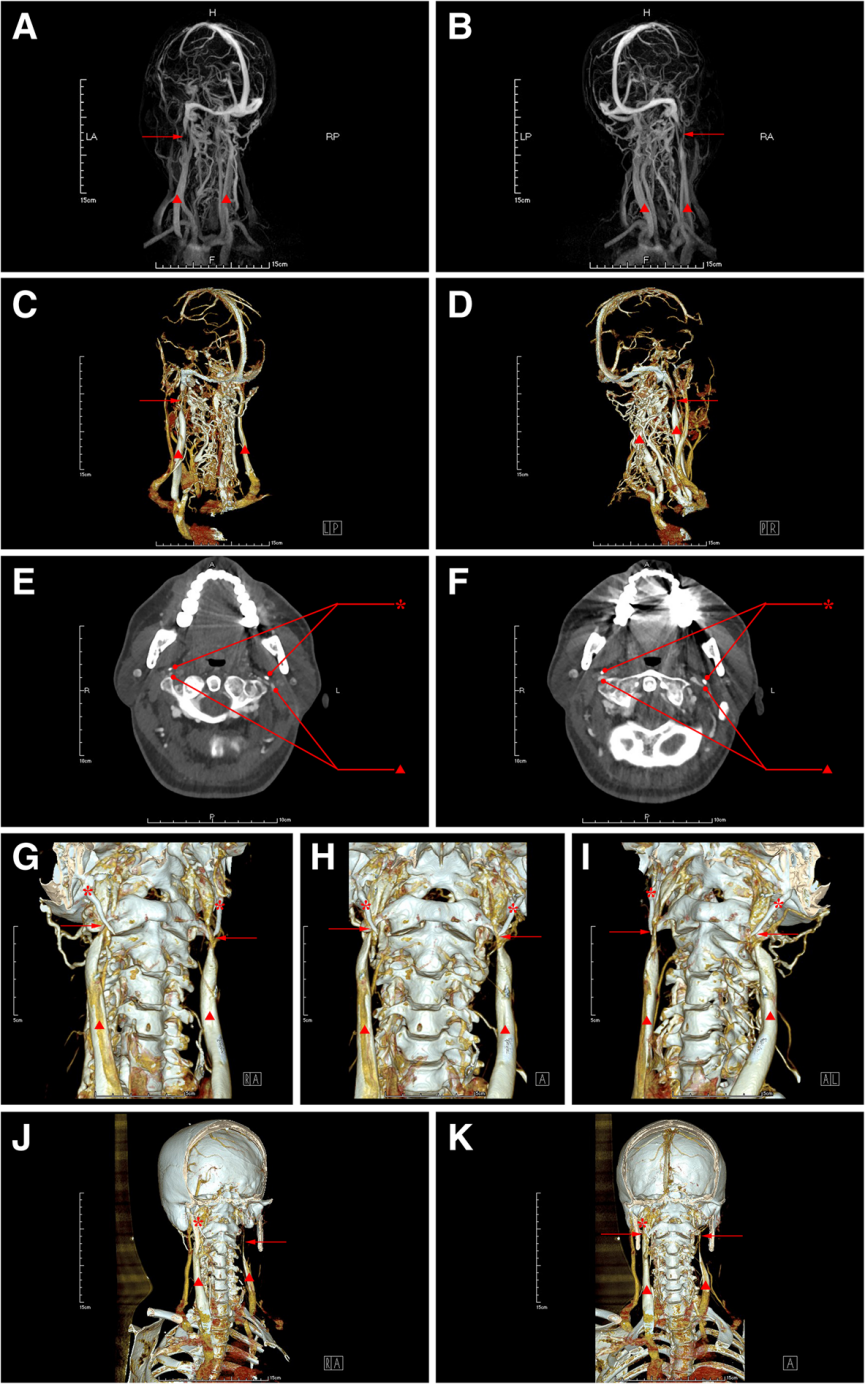
Images of Contrast-MRV (**a-b**), 3D-CTV (**c-d**), axial CTV (**e-f**) and 3D-CTV with bone remodeling (**g-i**) before styloidectomy and 3D-CTV with bone remodeling (**j-k**) after styloidectomy in case 5. The red asterisk represents styloid process, the red triangle represents internal jugular vein and the red arrow indicates the focal stenosis

Lumbar puncture showed the intracranial pressure (ICP) was 220mmH_2_O. Jugular vein ultrasound indicated stenosis at bilateral IJV-J3 segment. Contrast-MRV (Fig. 5a-b) and 3D-CTV (Fig. 5c-d) revealed the stenosis at bilateral IJV-J3 segment and dilated vertebral venous plexus. DSA identified a severe stenosis at bilateral IJV-J3 segment and occlusion in the superior trunk of middle cerebral artery. Axial CTV (Fig. 4e-f) and 3D-CTV (Fig. 4g-i) displayed that the J3 segment of bilateral IJV was compressed by styloid process and transverse process of C1 vertebra.

After underwent styloidectomy in the left side, symptoms of the patient were partially attenuated, the CTV follow-up images at the 5th day post- styloidectomy revealed an absence of left styloid process and left transverse process of C1 vertebra (Fig. 5k-l), and both the results of Jugular vein ultrasound and CTV images (Fig. 5k-l) followed-up at the 5th day post-styloidectomy showed that the stenosis at left IJV-J3 segment still existed, whereas, symptoms of the patient were obviously attenuated at the 11st day after styloidectomy and the blood flow in left IJV Jugular vein was partly improved confirmed by ultrasound. At 3 months follow-up after styloidectomy, the jugular vein blood flow in left IJV was turned to normal confirmed by ultrasound follow-up.

## Discussion and conclusions

The etiologies of IJVS so far reported, include congenital disorders, thrombosis, stenosis caused by venous wall disorder, demyelinating diseases and extrinsic compression [3, 11–14]. Congenital disorders include aplasia or hypoplasia of IJV. Internal jugular vein thrombosis (IJVT) can occur spontaneously or as a complication of surgical operation, IJVS, head and neck infection, malignant tumor, polycythemia, hyperhomocysteinemia, neck massage or intravenous drug abuse [7, 13, 15]. Extrinsic compression accounts for a large proportion of IJVS. Currently recognized causes of extrinsic compression are styloid process, digastric muscle, arteries, sternocleidomastoid muscle and thoracic outlet syndrome [11, 14].

It is reported that IJVS patients presents with intracranial hypertension (IH) and high-pressure gradient across stenosis [1, 16]. Non- thrombosis and non- extrinsic compression induced IJVS and its symptoms can be corrected by stenting [1]. In case 4 in this study, the symptoms were resolved by stenting and reoccurred after restenosis of IJV, indicating that IJVS is the causation of these symptoms. In case 5 in this study, the symptoms were disappeared after styloidectomy. Dashti et al. [16] also reported two patients with central venous outflow obstruction secondary to lateral tubercle of C1 and styloid process. The dominant jugular vein was decompressed and the symptoms of intracranial hypertension were resolved after the styloid process was resected. It is demonstrated that styloid oppression is the causation of IJVS mediated symptoms.

It demonstrated that jugular vein ultrasound is more sensitive in screening IJVS [17], which is recommended as the preferred test. It is reported that MRV is preferable to CTV in terms of high accuracy of diagnosis [18]. The present cases showed that CTV has the advantage of revealing the extrinsic compression when compared with jugular vein ultrasound, MRV and DSA. The first 3 cases showed that conservative treatments may have no efficacy to styloid oppression-induced IJVS. Case 4 indicated that stenting may be also inappropriate. Case 5 revealed that styloidectomy may be effective in treating styloid oppression-induced IJVS. However, further clinical trials are required to give a solid conclusion.

Eagle syndrome (ES) is a set of symptoms associated with an elongated styloid process [4, 6]. ES is classified into classic syndrome and Stylo-carotid syndrome. Classic syndrome describes compression of glossopharyngeal nerve by styloid process. Styloid-carotid syndrome refers to stenosis or dissection of internal carotid artery caused by compression of styloid process. This paper reports impingement on IJV by styloid process, causing venous reflux obstruction. We propose that this syndrome should be named as the stylo-jugular ES.

This cases series study suggests that IJVS is a pathological condition, which should not be ignored, and extrinsic compression, especially by styloid process, is one of the important etiologies of IJVS. CTV may be a useful tool for confirming the diagnosis of stylo-jugular ES. More studies are required to determine whether styloidectomy serves as a promising therapeutic strategy in styloid oppression-induced IJVS patients with an elevated trans-stenotic venous pressure gradient and symptoms plausibly related to raised cerebral venous pressure.

## Data Availability

The data that support the findings of this study are available from the corresponding author via E-mail upon reasonable request.

## Abbreviations

3D: Three dimensional
BBTI: Black-blood thrombus imaging
CT: Computed tomography
CTV: Computed tomography venography
DSA: Digital subtraction angiography
ES: Eagle syndrome
IJV: Internal jugular vein
IJVS: Internal jugular vein stenosis
IJVT: Internal jugular vein thrombosis
MRI: magnetic resonance imaging
MRV: Magnetic resonance venography
MS: Multiple sclerosis
TCCD: Transcranial color doppler
